# Unique MicroRNA and mRNA Interactions in *EGFR*-Mutated Lung Adenocarcinoma

**DOI:** 10.3390/jcm7110419

**Published:** 2018-11-06

**Authors:** Sophia Subat, Kentaro Inamura, Hironori Ninomiya, Hiroko Nagano, Sakae Okumura, Yuichi Ishikawa

**Affiliations:** 1 Division of Pathology, The Cancer Institute; Department of Pathology, The Cancer Institute Hospital, Japanese Foundation for Cancer Research, 3-8-31 Ariake, Koto-ward, Tokyo 135-8550, Japan; sophia.subat@jfcr.or.jp (S.S.); hironori.ninomiya@jfcr.or.jp (H.N.); hiroko.nagano@jfcr.or.jp (H.N.); ishikawa@jfcr.or.jp (Y.I.); 2 Department of Thoracic Surgical Oncology, The Cancer Institute Hospital, Japanese Foundation for Cancer Research, 3-8-31 Ariake, Koto-ward, Tokyo 135-8550, Japan; sokumura@jfcr.or.jp

**Keywords:** driver mutation, EGFR, integrative association, messenger RNA, molecular-targeted therapy, noncoding RNA, non-small-cell lung carcinoma (NSCLC), precision medicine, tumorigenesis, tyrosine kinase inhibitor

## Abstract

The *EGFR* gene was one of the first molecules to be selected for targeted gene therapy. *EGFR*-mutated lung adenocarcinoma, which is responsive to *EGFR* inhibitors, is characterized by a distinct oncogenic pathway in which unique microRNA (miRNA)–mRNA interactions have been observed. However, little information is available about the miRNA–mRNA regulatory network involved. Both miRNA and mRNA expression profiles were investigated using microarrays in 155 surgically resected specimens of lung adenocarcinoma with a known *EGFR* mutation status (52 mutated and 103 wild-type cases). An integrative analysis of the data was performed to identify the unique miRNA–mRNA regulatory network in *EGFR*-mutated lung adenocarcinoma. Expression profiling of miRNAs and mRNAs yielded characteristic miRNA/mRNA signatures (19 miRNAs/431 mRNAs) in *EGFR*-mutated lung adenocarcinoma. Five of the 19 miRNAs were previously listed as *EGFR*-mutation-specific miRNAs (i.e., miR-532-3p, miR-500a-3p, miR-224-5p, miR-502-3p, and miR-532-5p). An integrative analysis of miRNA and mRNA expression revealed a refined list of putative miRNA–mRNA interactions, of which 63 were potentially involved in *EGFR*-mutated tumors. Network structural analysis provided a comprehensive view of the complex miRNA–mRNA interactions in *EGFR*-mutated lung adenocarcinoma, including DUSP4 and MUC4 axes. Overall, this observational study provides insight into the unique miRNA–mRNA regulatory network present in *EGFR*-mutated tumors. Our findings, if validated, would inform future research examining the interplay of miRNAs and mRNAs in *EGFR*-mutated lung adenocarcinoma.

## 1. Introduction

Globally, lung cancer is the leading cause of cancer-related deaths, resulting in more than one million deaths annually [1]. Lung cancer can be generally classified into small-cell lung carcinoma (SCLC; 20% of all lung cancers) and non-SCLC (NSCLC; 80%), with adenocarcinoma representing the most prevalent subtype [2]. In lung adenocarcinoma, various mutations that drive cancer progression have emerged as druggable molecular targets [3,4,5,6,7]. Among them, *EGFR* mutation is the most prevalent genetic alterations, identifiable in 10−15% of Western and 30−40% of Asian populations [8,9,10,11]. Due to its frequent mutation, *EGFR* was one of the first molecules selected for targeted gene therapy [12]. Recent high-throughput techniques have substantially increased our knowledge of oncogenic mechanisms involved in *EGFR*-mutated adenocarcinoma. However, the mechanisms underlying its initiation and progression are not fully understood [3,8,11].

MicroRNAs (miRNAs) are small noncoding RNAs composed of 18–25 nucleotides. Over the past decade, approximately 2000 human miRNAs have been identified [13]. Individual miRNAs have been shown to target a number of mRNAs and negatively regulate their expression by either inhibiting translation or inducing mRNA degradation, as each mRNA is regulated by multiple miRNAs [14,15,16,17,18,19]. Complex interactions between miRNAs and mRNAs play crucial roles in a variety of cellular processes, such as cellular differentiation, development, and tumorigenesis [16,17,18,19,20,21]. Aberrantly expressed miRNAs act either as tumor suppressors or enhancers in a context-dependent manner. Previous research has shown that miRNA–mRNA interactions differentially regulate the oncogenic processes involved in lung cancer according to the presence of driver mutations in the tumor [22,23,24,25,26,27]. In *EGFR*-mutated cancers, individual miRNA–mRNA interactions or miRNA/mRNA expression signatures have been characterized. However, no study has performed integrative analysis to investigate the complex miRNA–mRNA network in *EGFR*-mutated lung adenocarcinoma. Little information is available about the miRNA–mRNA regulatory network involved in this type of tumor.

In this study, we examined 155 surgically resected specimens of lung adenocarcinoma with a known *EGFR* mutation status (52 mutated and 103 wild-type cases) to identify the complex miRNA–mRNA regulatory network involved in this type of tumor. Using microarray and bioinformatics analyses, this study is the first comprehensive report of the miRNA–mRNA network present in *EGFR*-mutated lung adenocarcinoma.

## 2. Materials and Methods

### 2.1. Clinical Samples

We obtained lung adenocarcinoma tissue from 155 Japanese patients who underwent surgery at The Cancer Institute Hospital, Japanese Foundation for Cancer Research (JFCR), Tokyo, Japan, between April 1995 and January 2002. Informed consent was obtained from all patients, and the study protocol was approved by the Institutional Review Board of JFCR on 1 February 2018 (ethics code: 2012-1042). All tumor samples were dissected and snap-frozen in liquid nitrogen within 20 min of removal and stored at −80 °C until RNA extraction.

### 2.2. EGFR Mutation

For the mutational analysis of *EGFR*, DNA was extracted from fresh tumor specimens using a standard proteinase K digestion and phenol–chloroform extraction. The four exons that encode the tyrosine kinase domain of the *EGFR* gene (exons 18 to 21) were examined. We performed TaqMan^TM^ SNP Genotyping Assays (Applied Biosystems, Foster City, CA, USA) not only for exons 18 (G719X) and 21 (L858R and L861Q), but also for exon 20 (S768I and T790M) according to the manufacturer’s instructions. Fragment analyses were conducted on the exon 19 deletion and exon 20 insertion, as previously described [28]. To investigate associations between *EGFR* mutations and clinicopathological factors, we used the Fisher’s exact test. All two-sided *p* values less than 0.05 were considered statistically significant.

### 2.3. RNA Extraction

Total RNAs were extracted from frozen tissue using miRNeasy Mini Kit (Qiagen, Hilden, Germany), according to the manufacturer’s instructions. RNA concentration, purity, and integrity number (RIN) were measured using the 2100 Bioanalyzer (Agilent Technologies, Palo, CA, USA). Only samples with an RIN greater than 2.0 were selected for microarray hybridization.

### 2.4. Expression Analysis of MicroRNAs

One-hundred nanograms of total RNA was labeled with cyanine-3-pCp, and then hybridized to an unrestricted human miRNA 8 × 60K microarray (release 19.0; Design ID 046064, Agilent Technologies), which covers 2042 human miRNAs, using Agilent’s miRNA Complete Labeling Reagent and Hybridization Kit (5190-0456, Agilent Technologies). After hybridization, the arrays were washed and scanned at high resolution using the Agilent Microarray Scanner System (G2565CA, Agilent Technologies).

Raw miRNA expression data were processed using the Bioconductor package AgiMicroRna (Processing and Differential Expression Analysis of Agilent microRNA chips; Agilent Technologies) in R environment, version 3.5 [29,30]. In AgiMicroRna, the linear model features implemented in the limma package were used to assess differential gene expression [31,32]. The miRNA microarray data were normalized using the Robust Multi-Array Average approach, whereas undetected probes were flagged and filtered if detected in fewer than one-half of the array replicates. The miRNA datasets are accessible through Gene Expression Omnibus (GEO) accession number GSE119267. To identify differentially expressed miRNAs according to their *EGFR* mutation status, the limma package was used; miRNAs with *p* values less than 0.05 and exhibiting differential expression according to their *EGFR* mutation status were selected.

### 2.5. Expression Analysis of Messenger RNAs

Fifty nanograms of total RNA was reverse-transcribed into cDNA and labeled using the Low Input Quick Amp Labeling Kit (5190-2305, Agilent Technologies), following the manufacturer’s instructions. The cDNAs were hybridized to the SurePrint G3 Human Gene Expression 8 × 60K Microarray (Design ID 028004, Agilent Technologies). After hybridization, the arrays were washed using the Gene Expression Wash Buffer Kit (Agilent Technologies), according to the manufacturer’s instructions. Scanning of the arrays was carried out using the Agilent Micro Scanner System (G2565CA, Agilent Technologies). Images and data were obtained using Agilent Feature Extraction Software, version 10.10.1.1 (Agilent Technologies).

Raw mRNA expression data were imported into R software, version 3.5. Background corrections were performed using the normexp method with an offset of 50, and quantiles were used for between-array normalization. Filtering of both controls and probes with low expression levels was performed by calculating the 95 percentiles of the negative control probes for each array. The datasets are accessible through GEO accession number GSE119268. To identify differentially expressed mRNAs according to their *EGFR* status, the limma package was used. Messenger RNAs with significant differences were selected with a fold change (FC) greater than 1.5 and a *p* value of less than 0.05.

### 2.6. Integrative Analysis

Integrated analyses of the miRNA and mRNA expression profiles were then carried out using differentially expressed miRNAs and mRNAs. MiRNA–mRNA correlations were identified using the miRComb package [33]. Pearson correlation coefficients between a particular miRNA and its predicted target mRNAs were computed and matched by target prediction using three databases: TargetScan, microCosm (formerly miRBase Targets), and miRDB [17,34,35]. Because miRNAs function as negative regulators, up- and downregulated miRNAs induce down- and upregulation of target mRNAs, respectively. We selected miRNA–mRNA pairs that were correlated in a negative manner (*p* < 0.01) and appeared in at least one of the three databases. The network was constructed and visualized with Cytoscape, version 3.6.1 (http://www.cytoscape.org). *p* values from the Pearson correlation estimates were corrected for multiple testing using the Benjamini–Hochberg method.

### 2.7. Functional Analysis

To identify the Kyoto Encyclopedia of Genes and Genomes (KEGG) pathways associated with select miRNAs, we used the union-of-pathways option of the bioinformatics prediction tool DNA Intelligent Analysis (DIANA)-miRPath software, version 3.0 [36]. To identify the biological pathways enriched by select mRNAs, we used the annotation tools from the Database for Annotation, Visualization, and Integrated Discovery (DAVID) Bioinformatics Resources, version 6.8 and selected categories with a *p* value less than 0.05 and a fold enrichment greater than 2 [37].

## 3. Results

### 3.1. Design of the Study and Clinicopathological Characteristics 

**Figure 1:**
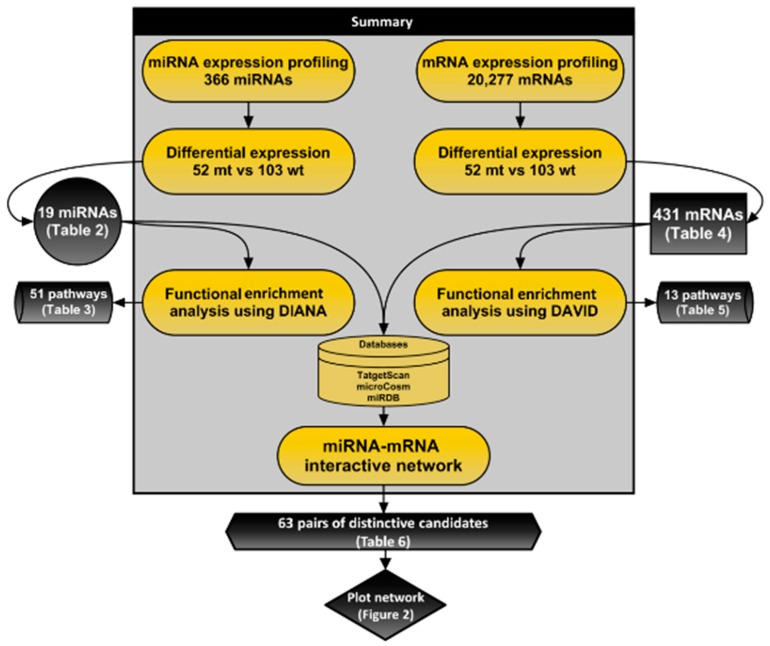
Flow diagram for predicting the target relationship between miRNA–mRNA pairs.

An overview of this study is outlined in Figure 1. We examined 155 surgically resected specimens of lung adenocarcinoma with a known *EGFR* mutation status (52 mutated and 103 wild-type cases). Table 1 summarizes the clinicopathological characteristics of the patients, stratified by *EGFR* status. *EGFR* mutation was associated with a no-to-moderate smoking history (pack-years < 40; *p* = 0.02) and a well-differentiated tumor (*p* < 0.0001).

### 3.2. Expression Profiling and Functional Analysis of MicroRNAs

In an effort to identify miRNA expression signatures in *EGFR*-mutated tumors, we performed expression profiling of miRNAs. After filtering, the expression levels of 19 miRNAs were found to differ between the *EGFR*-mutated and wild-type groups, which consisted of 16 upregulated and three downregulated miRNAs (Table 2). Of note, five of these miRNAs were included in the previously reported 17-miRNA signature in *EGFR*-mutated lung adenocarcinoma: miR-532-3p, miR-500a-3p, miR-224-5p, miR-502-3p, and miR-532-5p [38].

Using DIANA-miRPath software, we investigated the characteristic biological processes in *EGFR*-mutated tumors based on miRNA expression. A total of 51 miRNA-involved processes were identified by a functional enrichment analysis (KEGG pathways; *p* < 0.05, false discovery rate corrected). Supplementary Table S1 lists the characteristic biological processes enriched by 51 miRNAs that were differentially expressed according to *EGFR* mutation status. The top 30 biological pathways included the “Hippo signaling pathway (hsa04390)” (*p* = 2.38E–08), “pathways in cancer (hsa05200)” (*p* = 4.33E–05), and “non-small-cell lung cancer (hsa05223)” (*p* = 3.75E–03), as shown in Table 3. “Pathways in cancer” was enriched in miRNAs, which included the five abovementioned concordant miRNAs [38].

### 3.3. Expression Profiling and Functional Enrichment Analysis of Messenger RNAs

Using data from mRNA microarrays, we conducted expression profiling and functional enrichment analysis to identify mRNA expression signatures and enriched biological processes. After filtering, we identified 431 differentially expressed mRNAs that consisted of 270 upregulated and 161 downregulated mRNAs in *EGFR*-mutated tumors (*p* < 0.05 and FC > 1.5), as listed in Supplementary Table S2. A number of these mRNAs are known to be deregulated in NSCLC, including *EGFR*-mutated lung adenocarcinoma [39,40,41,42,43]. For example, our core mRNA signature included tumor-suppressor genes CDK6 and RB1 [39,40,41,42]. Moreover, the mRNA expression signature included DUSP4, EGFR, TNFRSF10B, and LRRC31, all of which were previously reported to be deregulated in *EGFR*-mutated tumors [43]. Table 4 lists the top 20 mRNAs that were differentially expressed between *EGFR*-mutated and wild-type tumors.

To investigate the potential biological functional relevance of the differentially expressed mRNAs, we performed functional enrichment analysis using DAVID pathways. Table 5 lists the top 13 KEGG pathways enriched by the 431 mRNAs that were differentially expressed according to *EGFR* status, providing an idea of which pathways were significantly enriched in *EGFR*-mutated tumors. Highly enriched pathways included “pathways in cancer” (*p* = 4.07E–03), “Wnt signaling pathway” (*p* = 4.30E–02), “small-cell lung cancer” (*p* = 4.88E–02), and “non-small-cell lung cancer” (*p* = 4.17E–02). The “nonsmall-cell lung cancer” pathway was enriched by mRNAs, including EGFR, RB1, and CDK6, all known to be associated with tumorigenesis in NSCLC [3,42,44].

### 3.4. MiRNA-mRNA Interactive Network

**Figure 2:**
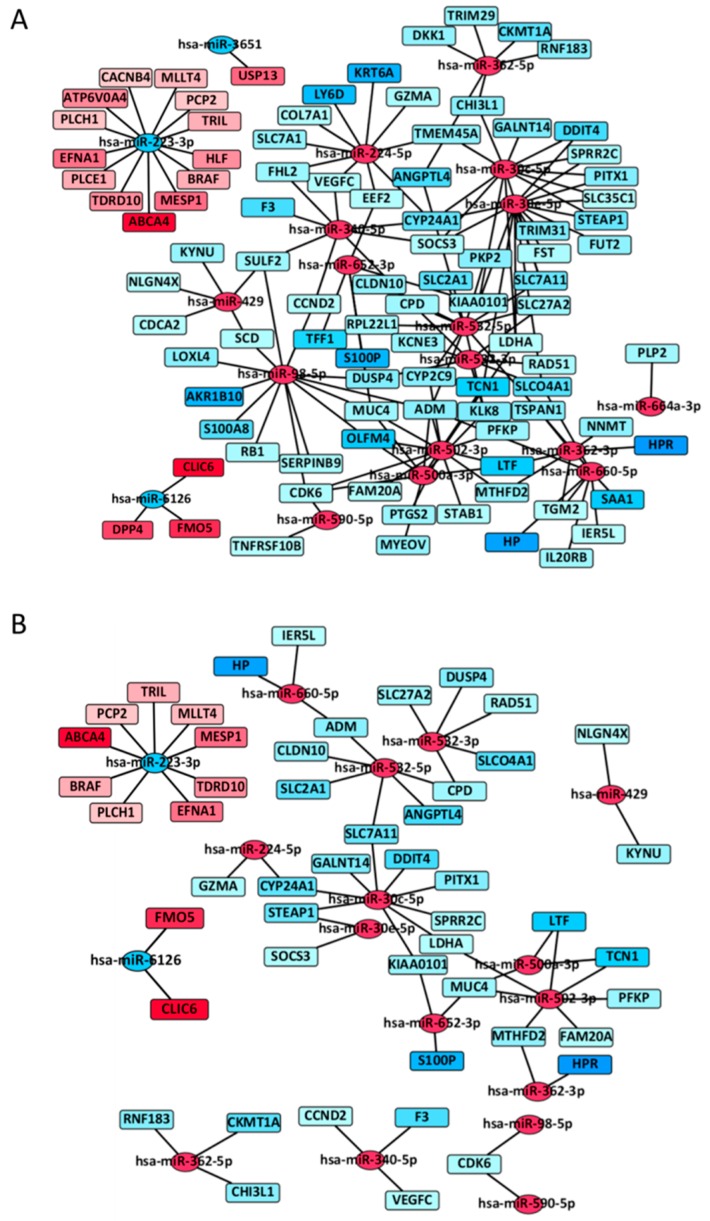
MiRNA–mRNA interactive network in *EGFR*-mutant lung adenocarcinoma. (**A**) Network of 149 miRNA-mRNA interactions in *EGFR*-mutated lung adenocarcinoma; (**B**) Network of 63 statistically significant miRNA–mRNA interactions in *EGFR*-mutated lung adenocarcinoma (*p* < 0.01). Circles and squares represent miRNAs and mRNAs, respectively. Red indicates an upregulated miRNA or mRNA, whereas blue indicates a downregulated miRNA or mRNA. A line indicates an miRNA–mRNA interaction. The color intensity is proportional to the fold change (continuous) of *EGFR*-mutated/wild-type lung adenocarcinoma.

To identify miRNA–mRNA interactions, we applied the miRComb package using miRNAs and mRNAs whose expression levels differed significantly in each dataset [45]. First, we identified a total of 149 miRNA–mRNAs pairs that showed negative correlations according to three miRNA target prediction databases: TargetScan, microcosm, and miRDB (Supplementary Table S3). To determine whether different miRNAs within an miRNA–mRNA signature interact with the same target genes, we performed network analysis using Cytoscape. Figure 2A shows all 149 miRNA–mRNA interactions, and Figure 2B represents the statistically significant miRNA–mRNA interactive network present in *EGFR*-mutated tumors (63 miRNA–mRNA interactions; *p* < 0.01). Table 6 lists the top 20 miRNA–mRNA pairs sorted by false discovery rate. Upregulated miR-30c-5p and downregulated miR-223-3p shared most of their target mRNAs. Interestingly, miR-532-5p and miR-532-3p, which belong to the same miR-532 family and are included in the enriched “pathways in cancer”, were located close to each other in the interactive network (Figure 2B). MUC4, the downregulation of which is associated with tumor progression in *EGFR*-mutated lung adenocarcinoma, was targeted by three miRNAs: miR-500a-3p, miR-502-3p, and miR-652-3p [46]. (Figure 2B and Supplementary Table S4).

## 4. Discussion

We examined surgically resected cases of *EGFR*-mutated (52 cases) and wild-type (103 cases) lung adenocarcinoma, and identified the unique miRNA–mRNA regulatory network present in *EGFR*-mutated tumors (Figure 1). Our findings, if validated, would identify a list of miRNA–mRNA interactions that could be used to understand the molecular pathogenesis of *EGFR*-mutated lung adenocarcinoma.

Lung cancer represents a group of molecularly heterogeneous tumors with different miRNA and mRNA expression signatures [14,16,22,23,47,48]. Numerous studies have previously reported genotype-specific miRNA and mRNA signatures in lung adenocarcinoma [38,43,49,50,51]. However, these signatures vary considerably and have few genes in common because of the different study populations, microarray platforms, and analytic methods utilized [52]. In *EGFR*-mutated lung adenocarcinoma, Bjaanæs et al. and Chitale et al. demonstrated characteristic miRNA and mRNA signatures, respectively; however, no studies have validated their results [38,43]; In the current study, we identified the 19-miRNA and 431-mRNA expression signatures in *EGFR*-mutated tumors. Typical miRNAs in the 19-miRNA signature include high expression levels of miR-532-3p, miR362-3p, miR-340-5p, miR-500a-3p, miR-224-5p, miR-502-3p, and miR-532-5p, and low expression levels of miR-223-3p, all related with tumorigenesis or tumor progression in NSCLC [38,49,51,53,54]. High expression levels of miR-224 and low expression levels of miR-223 have been previously reported to promote tumor progression in lung cancer [53,54]. Of note, five of the 19 miRNAs were also included in the previously reported 17-miRNA expression signature in *EGFR*-mutated lung adenocarcinoma [38]. Similarly, our mRNA expression signature in *EGFR*-mutated tumors included DUSP4, EGFR, TNFRSF10B, and LRRC3, all reportedly deregulated in *EGFR*-mutated lung adenocarcinoma [43]. Taken together, our results are concordant with previous findings, which enhance the credibility of our findings.

Accumulating evidence has led to the identification of characteristic oncogenic and biological processes in *EGFR*-mutated or -deregulated tumors [11,55,56,57]. *EGFR* aberrations activate multiple downstream pro-oncogenic signaling pathways and subsequently induce biological processes that are beneficial to cancer maintenance and progression, including chronic initiation, metabolic regulation, and cell-cycle regulation [58]. We identified the oncogenic or biological processes that were characteristically observed in *EGFR*-mutated lung adenocarcinoma on the basis of miRNA and mRNA expression levels. Interestingly, the miRNA expression signature indicates that the Hippo signaling pathway may be deregulated in *EGFR*-mutated tumors. The Hippo pathway is a kinase cascade stimulated by the *YAP1* oncogene, the activation of which is associated with resistance to EGFR inhibitors in *EGFR*-mutated lung adenocarcinoma [59,60,61]. The Hippo pathway appears to be characteristically deregulated in *EGFR*-mutated tumors and may thus serve as a therapeutic target. On the other hand, analysis of mRNA expression levels yielded the existence of the “pathways in cancer” and “non-small-cell lung cancer” pathways, which were enriched in *EGFR*-mutated tumors. Both of these pathways predictably lead to high expression levels of EGFR mRNA, given that *EGFR* amplification frequently promotes tumor invasion in *EGFR*-mutated lung adenocarcinoma [62]. The enriched “non-small-cell lung cancer” pathway included downregulated RB1 and CDK6, well-known tumor suppressors that regulate cell division and the cell cycle. Collectively, these results help define the mechanisms that underlie tumor initiation or progression in *EGFR*-mutated lung adenocarcinoma.

*EGFR*-mutated lung adenocarcinoma is orchestrated by complex miRNA–mRNA interactions. Much work has investigated miRNA/mRNA expression signatures and associations between a specific miRNA and target mRNAs in tumorigenic processes in *EGFR*-mutated tumors [11,14,16,17,18,23,48,63,64,65,66]. However, no study has comprehensively analyzed complex miRNA–mRNA interactions in *EGFR*-mutated tumors. Typical interactions included miRNA-532-3p/RAD51 and miRNA-532-3p/DUSP4 interactions. Zhong et al. reported that the mutated *EGFR* mediates the role of RAD51 in regulating radiation-induced cell-cycle arrest [67]. Chitale et al. reported that *EGFR*-mutated lung adenocarcinoma was strongly associated with low expression levels of DUSP4 [43]. These findings suggest that miR-532-3p functions as an oncogenic miRNA by downregulating the tumor suppressors RAD51 and DUSP4. Additionally, complex interactions between upregulated miR-500a-3p, miR-502-3p, and miR-652-3p, and downregulated MUC4 were identified in *EGFR*-mutated tumors. Downregulated MUC4 has been reported to induce tumor progression in conjunction with an *EGFR* mutation [46]. Taken together, the concordant results in this and previous studies help identify characteristic oncogenic pathways in *EGFR*-mutated lung adenocarcinoma.

This study had limitations. First, its observational nature precludes the determination of a causal association between an individual miRNA and its target mRNAs. Certain mechanisms of gene regulation might be due to indirect effects by other modulators, such as transcription factors. Therefore, further research is required to mechanistically validate the interactions predicted by our results. Second, we did not investigate *KRAS*, *ALK*, or other driver mutations. Therefore, it would be necessary to confirm that miRNA–mRNA interactions identified in this study are truly associated with *EGFR* status. Third, clinicopathological variables might confound miRNA–mRNA interactions. Finally, no validation study was performed. Nonetheless, our results are concordant with previous observations, which enhance the credibility of our findings [38,43]. Further investigations with a larger sample size and other races are warranted to confirm our findings.

## 5. Conclusions

In summary, we identified the unique miRNA–mRNA interactions in *EGFR*-mutated lung adenocarcinoma. To our knowledge, this is the first study to identify the unique miRNA–mRNA network involved in *EGFR*-mutated lung adenocarcinoma. Our findings, if validated, would inform future research examining the interplay between miRNAs and mRNAs in *EGFR*-mutated lung adenocarcinoma.

## Figures and Tables

**Table 1:** Clinicopathological characteristics of 155 cases of lung adenocarcinoma stratified by *EGFR* mutation status.

Variables	Number of Patients (%)	*EGFR*		*p* Values ^1^
		Mutation 52 (34%)	Wild-Type 103 (66%)	
Age (years)				0.30
<65	96 (62%)	29 (56%)	67 (65%)	
≥65	59 (38%)	23 (44%)	36 (35%)	
Gender				0.13
Males	77 (50%)	21 (40%)	56 (54%)	
Females	78 (50%)	31 (60%)	47 (46%)	
Smoking status				0.40
Never	81 (52%)	30 (58%)	51 (50%)	
Ever	74 (48%)	22 (42%)	52 (50%)	
Cumulative smoking				0.02
Pack-years < 40	123 (79%)	47 (90%)	76 (74%)	
Pack-years ≥ 40	32 (21%)	5 (10%)	24 (26%)	
Size (mm)				0.23
<30	91 (59%)	27 (52%)	64 (62%)	
≥30	64 (41%)	25 (48%)	39 (38%)	
Tumor differentiation				<0.0001
Well	60 (39%)	37 (72%)	23 (22%)	
Moderate–poor	95 (61%)	15 (29%)	80 (78%)	
Pathological stage				0.39
I	93 (60%)	34 (65%)	59 (57%)	
II–IV	62 (40%)	18 (35%)	44 (43%)	

^1^ The Fisher’s exact test was used to calculate *p* values.

**Table 2:** Nineteen miRNAs that were differentially expressed in *EGFR*-mutated compared to *EGFR* wild-type lung adenocarcinomas.

miRNA	FC ^1^	*p* Values ^2^	Up or Down
miR-532-3p	3.52	1.32E–04	Up
miR-362-3p	3.29	1.02E–04	Up
miR-340-5p	3.25	3.44E–03	Up
miR-500a-3p	2.93	1.32E–04	Up
miR-224-5p	2.83	1.49E–02	Up
miR-362-5p	2.82	1.04E–03	Up
miR-502-3p	2.65	1.66E–03	Up
miR-590-5p	2.37	1.33E–02	Up
miR-664a-3p	2.31	4.86E–02	Up
miR-652-3p	2.12	1.37E–02	Up
miR-532-5p	2.05	1.91E–03	Up
miR-429	1.92	1.68E–02	Up
miR-660-5p	1.70	1.14E–02	Up
miR-30e-5p	1.38	3.44E–03	Up
miR-30c-5p	1.34	3.09E–02	Up
miR-98-5p	1.34	3.27E–02	Up
miR-6126	−1.25	4.86E–02	Down
miR-3651	−1.36	4.57E–02	Down
miR-223-3p	−1.66	1.37E–02	Down

FC, fold change. ^1^ *EGFR*-mutated lung/*EGFR* wild-type lung adenocarcinoma; ^2^ *p* values of the Pearson correlation estimate were corrected for multiple testing (Benjamini–Hochberg method applied).

**Table 3:** Top 30 biological pathways enriched by 19 differentially expressed miRNAs in *EGFR*-mutated compared to *EGFR* wild-type lung adenocarcinoma.

KEGG Pathway	*p* Values	*n*^1^ of miRNAs
Hippo signaling pathway	2.38E–08	18
Pathways in cancer	4.33E–05	18
FoxO signaling pathway	2.38E–08	17
Signaling pathways regulating pluripotency of stem cells	3.84E–07	17
PI3K-Akt signaling pathway	2.44E–04	17
Chronic myeloid leukemia	4.43E–04	17
Glioma	9.11E–04	17
T-cell receptor signaling pathway	1.42E–03	17
Regulation of actin cytoskeleton	1.70E–03	17
Neurotrophin signaling pathway	2.80E–03	17
Prolactin signaling pathway	3.26E–03	17
Viral carcinogenesis	3.26E–03	17
MAPK signaling pathway	3.50E–03	17
Non-small cell lung cancer	3.75E–03	17
Thyroid hormone signaling pathway	3.79E–03	17
cAMP signaling pathway	4.95E–03	17
Melanoma	7.01E–03	17
cGMP-PKG signaling pathway	7.40E–03	17
Dorsoventral axis formation	1.99E–02	17
Wnt signaling pathway	2.59E–02	17
B cell receptor signaling pathway	2.80E–02	17
Ubiquitin mediated proteolysis	2.82E–02	17
TGF-beta signaling pathway	8.42E–08	16
Proteoglycans in cancer	1.36E–07	16
Transcriptional misregulation in cancer	3.26E–07	16
Pancreatic cancer	3.34E–05	16
Ras signaling pathway	8.22E–05	16
ErbB signaling pathway	1.12E–04	16
GABAergic synapse	3.59E–04	16
Focal adhesion	8.84E–04	16

KEGG, Kyoto Encyclopedia of Genes and Genomes. ^1^ *n*, number of miRNAs that enriched each biological pathway. Pathways with *p* value less than 0.05 and thresholds for predictions greater than 0.8 are listed with the number of affected genes.

**Table 4:** Top 20 mRNAs that were differentially expressed between *EGFR*-mutated compared to *EGFR* wild-type lung adenocarcinoma.

mRNA	FC ^1^	*p* Values ^2^	Up or Down
LRRC75B	2.35	2.77E–13	Up
EGFR	1.88	1.78E–11	Up
KIAA0319L	1.65	2.08E–10	Up
CA10	2.75	2.31E–10	Up
USP13	1.82	2.31E–10	Up
CECR2	1.65	2.31E–10	Up
TBXT	1.63	2.54E–10	Up
LCT	1.83	5.56E–09	Up
MYBPHL	3.44	6.00E–09	Up
GGTLC2	2.15	9.00E–09	Up
GGTLC1	2.77	1.47E–08	Up
FNDC10	2.51	1.74E–08	Up
ATP13A4	3.10	1.86E–08	Up
DDX21	−1.60	2.41E–08	Down
SCUBE2	2.38	2.67E–08	Up
APOH	3.24	1.40E–07	Up
SERPINA3	−3.89	1.49E–07	Down
GFRA3	2.85	1.58E–07	Up
MEGF6	1.88	1.58E–07	Up
SLC41A1	1.67	1.95E–07	Up

^1^*EGFR*-mutated lung/*EGFR* wild-type lung adenocarcinoma; ^2^ *p* value of the Pearson correlation estimate was corrected for multiple testing (Benjamini–Hochberg method applied).

**Table 5:** Biologic pathways enriched by 431 differentially expressed mRNAs in *EGFR*-mutated compared to *EGFR* wild-type lung adenocarcinomas.

KEGG_PATHWAY	*p* Values	*n* ^1^	Genes
Pathways in cancer	4.70E–03	19	COL4A4,FZD9,EGFR,CEBPA,COL4A3,FGFR3,PTGS2,BRAF,EGLN3,CDK6,RB1,BIRC3,DAPK2,RAD51,VEGFC,CBLC,FZD10,WNT3,SLC2A1
Protein digestion and absorption	1.00E–03	9	COL4A4,COL4A3,COL21A1,COL7A1,KCNK5,PRSS3,PRSS1,DPP4,KCNE3
Arachidonic acid metabolism	5.69E–04	8	AKR1C3,GPX2,PTGS2,CYP2B6,CYP2C9,ALOX15B,PLA2G1B,GGT1
Hepatitis C	3.64E–02	8	EGFR,OCLN,BRAF,CLDN3,SOCS3,CLDN2,CLDN10,OAS1
Wnt signaling pathway	4.30E–02	8	FZD9,FZD10,WNT3,DKK1,CCND2,VANGL2,BAMBI,DAAM2
Central carbon metabolism in cancer	3.69E–03	7	GLS2,EGFR,FGFR3,GLS,SLC2A1,PFKP,PGAM2
Small-cell lung cancer	4.88E–02	6	COL4A4,COL4A3,PTGS2,CDK6,RB1,BIRC3
Bladder cancer	1.50E–02	5	EGFR,FGFR3,BRAF,RB1,DAPK2
Non-small-cell lung cancer	4.17E–02	5	EGFR,BRAF,CDK6,RB1,ALK
Nicotinate and nicotinamide metabolism	2.96E–02	4	NT5M,ENPP3,QPRT,NNMT
Galactose metabolism	3.23E–02	4	AKR1B10,AKR1B1,PFKP,LCT
Fructose and mannose metabolism	3.82E–02	4	GMPPB,AKR1B10,AKR1B1,PFKP
Alanine, aspartate and glutamate metabolism	4.79E–02	4	GLS2,GLS,CPS1,RIMKLA

^1^*n*, number of mRNAs that enriched each biological pathway. Pathways with *p* value less than 0.05 and fold enrichment greater than 2 are listed with the number of affected genes.

**Table 6:** Top 20 significant miRNA–mRNA interaction pairs.

miRNA	FC_miRNA ^1^	mRNA	FC_mRNA ^1^	FDR
miR-502-3p	Up	MTHFD2	Down	1.00E–09
miR-502-3p	Up	LTF	Down	1.20E–07
miR-502-3p	Up	PFKP	Down	2.11E–07
miR-30c-5p	Up	GALNT14	Down	5.85E–07
miR-30c-5p	Up	STEAP1	Down	6.07E–07
miR-532-3p	Up	CPD	Down	7.25E–07
miR-502-3p	Up	LDHA	Down	9.39E–07
miR-223-3p	Down	BRAF	Up	1.36E–06
miR-6126	Down	CLIC6	Up	1.92E–06
miR-30c-5p	Up	KIAA0101	Down	3.04E–06
miR-532-5p	Up	CPD	Down	3.19E–06
miR-532-3p	Up	RAD51	Down	5.83E–06
miR-532-3p	Up	DUSP4	Down	6.70E–06
miR-500a-3p	Up	LTF	Down	1.17E–05
miR-502-3p	Up	MUC4	Down	1.71E–05
miR-223-3p	Down	PLCH1	Up	1.79E–05
miR-532-5p	Up	ANGPTL4	Down	1.83E–05
miR-532-5p	Up	CLDN10	Down	2.02E–05
miR-6126	Down	FMO5	Up	2.57E–05
miR-660-5p	Up	IER5L	Down	3.69E–05

FDR, false discovery rate. ^1^ *EGFR*-mutated lung/*EGFR* wild-type lung adenocarcinoma.

## Supplementary Materials

The following are available online at http://www.mdpi.com/2077-0383/7/11/419/s1, Table S1: Pathway mapping of differentially expressed miRNAs, Table S2: 431 mRNAs that were significant differentially expressed between EGFR-mutated and EGFR-wild tumors, Table S3: miRNA-mRNA interactions predicted by miRComb, Table S4: Characteristics of patients and tumors included in the microarray.
